# Bmi-1 Regulates Snail Expression and Promotes Metastasis Ability in Head and Neck Squamous Cancer-Derived ALDH1 Positive Cells

**DOI:** 10.1155/2011/609259

**Published:** 2010-09-27

**Authors:** Cheng-Chia Yu, Wen-Liang Lo, Yi-Wei Chen, Pin-I Huang, Han-Shui Hsu, Ling-Ming Tseng, Shih-Chieh Hung, Shou-Yen Kao, Charn-Jung Chang, Shih Hwa Chiou

**Affiliations:** ^1^Institute of Oral Biology and Biomaterial Science, Chung-Shan Medical University, Taichung 40201, Taiwan; ^2^Department of Dentistry, Chung Shan Medical University Hospital, Taichung 40201, Taiwan; ^3^Division of Oral and Maxillofacial Surgery, Department of Stomatology, Taipei Veterans General Hospital, Taipei 11217, Taiwan; ^4^Department of Medical Research and Education, Taipei Veterans General Hospital, Taipei 11217, Taiwan; ^5^Institute of Clinical Medicine, National Yang-Ming University, Taipei 112, Taiwan; ^6^Department of Surgery, Taipei Veterans General Hospital, Taipei 11217, Taiwan; ^7^Department of Pharmacy Practice, Tri-Service General Hospital and 114 National Defense Medical Center, Taipei, Taiwan

## Abstract

Recent studies suggest that ALDH1 is a putative marker for HNSCC-derived cancer stem cells. However, the regulation mechanisms that maintain the stemness and metastatic capability of HNSCC-ALDH1^+^ cells remain unclear. Initially, HNSCC-ALDH1^+^ cells from HNSCC patient showed cancer stemness properties, and high expression of Bmi1 and Snail. Functionally, tumorigenic properties of HNSCC-ALDH1^+^ cells could be downregulated by knockdown of Bmi-1. Overexpression of Bmi-1 altered in expression property ALDH1^−^ cells to that of ALDH1^+^ cells. Furthermore, knockdown of Bmi-1 enhanced the radiosensitivity of radiation-treated HNSCC-ALDH1^+^ cells. Moreover, overexpression of Bmi-1 in HNSCC-ALDH1^−^ cells increased tumor volume and number of pulmonary metastatic lesions by xenotransplant assay. Importantly, knock-down of Bmi1 in HNSCC-ALDH1^+^ cells significantly decreased distant metastases in the lungs. Clinically, coexpression of Bmi-1/Snail/ALDH1 predicted the worst prognosis in HNSCC patients. Collectively, our data suggested that Bmi-1 plays a key role in regulating Snail expression and cancer stemness properties of HNSCC-ALDH1^+^ cells.

## 1. Introduction

Head and neck squamous cell carcinoma (HNSCC), including oral squamous cell carcinoma (OSCC), is the sixth most prevalent type of malignancy worldwide and accounts for approximately 8% to 10% of all cancers in Southeast Asia [1, 2]. HNSCC-related mortality is mainly caused by cervical lymph node metastasis, and occasionally by distant organ metastasis [3]. 

The epithelial-mesenchymal transition (EMT) is a process in which epithelial cells lose their polarity and adopt a mesenchymal phenotype [4]. This process is thought to be a critical step in the induction of tumor metastasis and malignancy [5]. Mani et al. demonstrated that induction of EMT results in cells that have stem cell properties and generates cells with properties similar to breast cancer stem cells [6]. Snail, a member of the zinc-finger transcription factor family, is one of the master regulators that promotes EMT and mediates invasiveness as well as metastasis in many different types of malignant tumors [7, 8]. The aldehyde dehydrogenase (ALDH) family of enzymes is comprised of cytosolic isoenzymes that oxidize intracellular aldehydes and contribute to the oxidation of retinol to retinoic acid in early stem cell differentiation [9]. Recently, ALDH has been reported to be a unique marker of head and neck cancer stem cells (CSC) [10, 11]. ALDH1 was also found to co-localize with other CSCs-related markers, including MMP-9, CD44, and CK14, at the invasive front of the tumor [12]. We previously reported the isolation of ALDH1-positive cells from patients with HNSCC [13]. These HNSCC-ALDH1^+^ cells displayed the radioresistance and represented a reservoir of cells that have the proliferative potential to generate tumors [13]. ALDH1^+^-lineage cells underwent EMT and endogenously co-expressed Snail [13]. These findings suggested that Snail expression may regulate the tumorigenesis, radiochemoresistance, and cancer stem cell properties of malignant HNSCC tumors [13]. However, the molecular mechanisms involved in mediating metastasis and tumor malignancy of HNSCC-CSC through the regulation of Snail remain unknown.

Bmi-1 is a member of the Polycomb (PcG) family of transcriptional repressors that mediate gene silencing by regulating chromatin structure [14]. Bmi-1 is essential for maintaining the ability of neural, hematopoietic, and intestinal stem cells to self-renew [15–17]. Bmi-1 was identified as a proto-oncogene that cooperates with MYC to promote the generation of lymphoma [18]. Bmi-1 also inhibited MYC-induced apoptosis by repressing the Cdkn2a locus [19]. Additionally, Bmi-1 has been verified as a predictor of prognosis in bladder cancer [20], prostate cancer [21], brain cancer [22, 23], breast cancer [24], pancreatic cancer [25], and lung cancer [26]. Bmi-1 has been demonstrated to play a role in the tumorigenesis of HNSCC [27, 28]. Bmi-1 has also been reported to be involved in tumor metastasis [29, 30]. Recently, an elegant study by Song et al. showed that Bmi-1 can directly promote EMT and malignancy in nasopharyngeal carcinoma by regulating Snail [31]. The goal of this study was to clarify the relationship between Bmi-1, Snail, and ALDH1 in HNSCC or HNSCC-associated CSC and the involved molecular mechanisms.

## 2. Materials and Methods

### 2.1. Isolation and Cultivation of HNSCC-Derived ALDH1^+^ and ALDH1^−^ Cells from HNSCC Patients

This study followed the tenets of the Declaration of Helsinki. All samples were obtained after patients provided informed consent. The study was approved by the Institutional Ethics Committee/Institutional Review Board of Taipei Veterans General Hospital. The information of HNSCC patients has been previously described in Table 1. The dissociated cells from the samples of HNSCC patients were suspended at 1 × 10^6^ cells/mL in 37°C DMEM supplemented with 2% FCS. The identification of aldehyde dehydrogenase 1 (ALDH1) positive HNSCC cells was carried out using the Aldefluor assay (StemCell Technologies, Durham, NC, USA) and fluorescence-activated cell sorting. Cells were suspended in ALDEFLUOR assay buffer containing ALDH substrate (BAAA, 1 *μ*mol/l per 1 × 10^6^ cells) and incubated for 40 min at 37°C. As a negative control, for each sample of cells, an aliquot was treated with 50 mmol/l diethylaminobenzaldehyde (DEAB), a specific ALDH inhibitor. The sorting gates were established using the cells stained with PI only as a negative control; the ALDEFLUOR-stained cells treated with DEAB and staining with a secondary antibody alone to test for viability. HNSCC-ALDH1^+^ cells were cultured in a medium consisting of serum-free DMEM/F12 (Gibco-BRL, Gaithersburg, MD), N2 supplement (R and D Systems Inc., Minneapolis), 10 ng/mL bFGF (R and D Systems), and 10 ng/mL EGF (R and D Systems) [13, 32].

### 2.2. Quantitative Real-Time RT-PCR

Briefly, total RNA (1 *μ*g) of each sample was reverse-transcribed using 0.5 *μ*g oligo dT and 200 U Superscript II RT (Invitrogen). The primer sequences for real-time RT-PCR were listed in Table 2. The amplification was carried out in a total volume of 20 *μ*L containing 0.5 *μ*mol·L^−1^ of each primer, 4 mmol·L^−1^ MgCl2, 2 *μ*L LightCyclerTM-FastStart DNA Master SYBR green I (Roche Molecular Systems, Alameda, CA), and 2 *μ*L of 1 : 10 diluted cDNA. PCR reactions were prepared in duplicate and performed using the following program: 95°C for 10 min, followed by 40 cycles of denaturation at 95°C for 10 sec, annealing at 55°C for 5 sec, and extension at 72°C for 20 sec. Standard curves (cycle threshold values versus template concentration) were prepared for each target gene and for the endogenous reference gene (GAPDH) for each sample. Quantification of unknown samples was performed using LightCycler Relative Quantification Software version 3.3 (Roche).

### 2.3. Knockdown and Overexpression of Bmi-1 with Lentivirus

The pLVRNAi vector was purchased from Biosettia Inc. (Biosettia, San Diego CA). The oligonucleotide 5′-AAAACCTAATACTTTCCAGATTGATTTGGAT CCAAATCAATCTGGAAAGTATTAGG-3′ targeting human Bmi-1 (NM_005180, nt 1061–1081) was synthesized and cloned into pLVRNAi to generate the lentiviral expression vector, pLVRNAi/sh-Bmi1. The lentiviral expression vector carrying Bmi-1 full-length cDNA, pLV/Bmi-1 was obtained from Biosettia Inc. pCMVΔR8.9 and pMD.G, expressing GAG-POL and the vesicular stomatitis virus envelope, respectively, were provided by the consortium (Academia Sinica, Taipei, Taiwan). The lentiviruses were generated by cotransfecting 5 × 10^6^ 293FT cells per 10 cm plate with lentiviral vector and packaging plasmids using Lipofectamine 2000 (LF2000, Invitrogen). Supernatants were collected 48 hours after transfection and filtered. The 48-hour posttransduction viral titers were determined by FACS. Subconfluent cells were infected with lentivirus at a multiplicity of infection of 5 in the presence of 8 *μ*g/mL polybrene (Sigma-Aldrich) [13, 33].

### 2.4. Microarray Analysis and Bioinformatics

Total RNA was extracted from cells using Trizol reagent (Life Technologies, Bethesda, MD, USA) and the Qiagen RNAeasy (Qiagen, Valencia, CA, USA) column for purification. Affymetrix HG U133 Plus 2.0 microarrays containing 54,675 probe sets for >47,000 transcripts and variants, including 38,500 human genes. A typical probeset contains eleven 25-mer oligo nucleotide pairs (a perfect match and a mismatch control). For microarray analysis, sample labeling, hybridization, and staining were carried out by Affymetrix standard protocol with *affyQCReport*. Probeset was normalized with loess method of all microarrays. The average linkage distance was used to assess the similarity between two groups of gene expression profiles as described below. The difference in distance between two groups of sample expression profiles to a third was assessed by comparing the corresponding average linkage distances (the mean of all pairwise distances (linkages) between members of the two groups concerned). The error of such a comparison was estimated by combining the standard errors (the standard deviation of pairwise linkages divided by the square root of the number of linkages) of the average linkage distances involved. Classical multidimensional scaling (MDS) was performed using the standard function of the R program to provide a visual impression of how the various sample groups are related.

### 2.5. In Vivo Tumor Growth and Metastasis

All procedures involving animals were in accordance with the institutional animal welfare guidelines of Taipei Veterans General Hospital. Eight-week-old SCID mice and/or nude mice (BALB/c strain) were injected with 105 cells orthotopically. *In vivo *GFP imaging was performed using an illuminating device (LT-9500 Illumatool/TLS equipped with an excitation source (470 nm) and filter plate (515 nm)). Tumor size was measured with calipers and the tumor volume was calculated using the formula (Length × Width2)/2. The integrated optical density of green fluorescence intensity was captured and analyzed using Image Pro-plus software [33, 34]. 

### 2.6. Statistical Analysis

The Statistical Package of Social Sciences software (SPSS, Inc., Chicago, IL) was used for statistical analysis. An independent Student's *t*-test was used to compare the continuous variables between groups. The Kaplan-Meier procedure was used to calculate survival probability estimates. A log-rank test was used to compare the cumulative survival durations in different patient groups. The statistical significance level was set at 0.05 for all tests.

## 3. Results

### 3.1. HNSCC-Derived ALDH1-Positive Cells Displayed Tumorigenic and Stemness Properties

**Figure 1:**
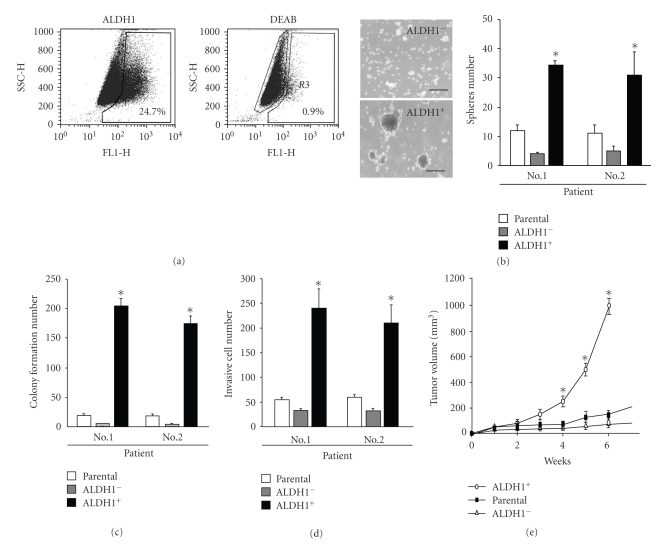
Isolation and Characterization of HNSCC-derived ALDH1-positive Cells. (a) Analyzing and sorting ALDH1^+^-positive and ALDH1^−^-negative from HNSCC tissues via FACScan. DEAB, an inhibitor of ALDH1, was used for negative control. (b) Evaluation of sphere body formation in the parental cells, ALDH1^−^ cells, and ALDH1^+^cells. Sphere bodies were counted after 1 week. The numbers of resultant colonies (c) and invasion cells (d) from parental cells, ALDH1^+^ cells, and ALDH1^−^cells were counted* in vitro*. (e) Macroscopic features of cells in a nude mouse at 6 weeks after xenotransplantation. Blue arrow indicates the site of injection of ALDH1^−^cells. Red arrow indicates the site of injection of ALDH1^+^cells. Yellow arrow indicates the site of injection of ALDH1^+^cells. *P < .05. Data shown here are the mean ± SD of three experiments.

Initially, parental, isolated ALDH1^+^, and ALDH1^−^ cells were isolated from tissue samples of six HNSCC patients using the Aldefluor assay and the fluorescence-activated cell sorting (FACS) analysis (Figure 1(a) and Table 1) [13, 35]. It has been reported that cancer stem-like cells can be cultured in suspension to generate floating spheroid-like bodies (SB) under serum-free medium with bFGF and EGF [36]. Interestingly, ALDH1^+^ increased higher tumor spheres-forming capability than that of ALDH1^−^ (Figure 1(b)). Furthermore, ALDH1^+^-derived spheres with regular 10% serum cultivation increased epithelial-attached cells and differentiation marker (CK18)(See Figure 1(a) in supplementary material available online at doi: 10.1155/2011/609259).To evaluate the enhancement of tumorigenicity of HNSCC-ALDH1^+^ cells, soft agar colony formation assays and Matrigel/Transwell-invasion and were examined. Compared with parental and ALDH1^−^, ALDH1^+^ derived from HNSCC Patients no.1 and no. 2 showed colony-forming ability and higher invasion activity (Figures 1(c) and 1(d)). To evaluate the *in vivo* tumor initiating capability of ALDH1^+^ and ALDH1^−^, we injected 1000, 3000, and 10^4^ cells into the neck of SCID mice. The results showed that 104 ALDH1^−^ did not induce tumor formation but 3,000 ALDH1^+^ from the HNSCC tissues of six patients in xenotransplanted mice all resulted in the generation of visible tumors 6 weeks after injection (Table 1).The results of xenotransplanted analysis further showed that ALDH1^+^ demonstrated higher abilities to induce tumor growth (Figure 1(e)). Lastly, serial xenotransplanted analysis suggested that ALDH1^+^ had in vivo self-renewal ability (Supplementary Figure 1(b)). Based on these findings, the ALDH1^+^-lineage cells isolated from HNSCC patients presented the significant tumor-initiating abilities, especially in ALDH1^+^ cells from patients no. 1 and no. 2. Real-time RT-PCR data demonstrated that the stemness and EMT-related genes (especially in Bmi-1 and Snail) were significantly activated in HNSCC ALDH1^+^ (Table 2 and data not shown).

### 3.2. Knockdown of Bmi-1 in HNSCC-ALDH1^+^ Cells Down-Regulates Snail and Lessens in vitro Tumorigenicity

**Figure 2:**
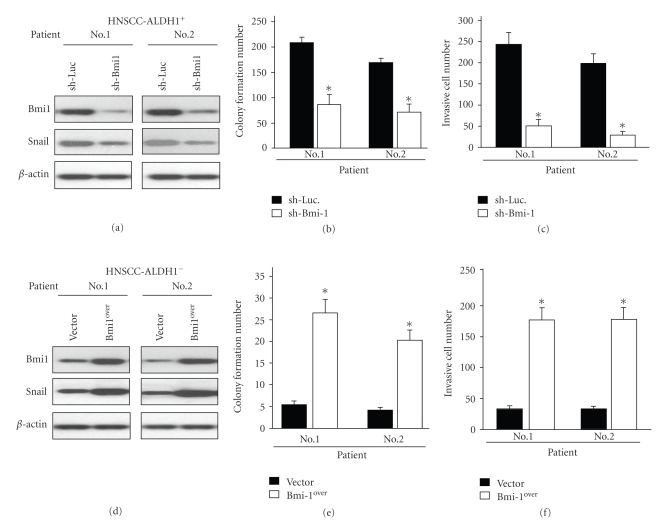
Overexpression of Bmi-1 in HNSCC-ALDH1^−^ cells or knockdown of Bmi-1 in HNSCC-ALDH^+^ cells modulates Snail expression and tumorigenicity* in vitro*. (a) Down-regulation of Bmi-1 mediated by lentiviral shRNA and expression of Snail and ALDH1 in HNSCC-ALDH1^+^ cells was analyzed by western blot. Colony formation (b) and migration/invasion ability (c) of shLuc.-expressing and shBmi-1-expressing HNSCC-ALDH1^+^cells was determined. (d) Total protein was prepared from control GFP–expressing andBmi-1-overexpressing HNSCC-ALDH1^−^ cells and analyzed by immunoblotting with anti-Bmi-1, anti-Snail, anti-ALDH1, or anti-GAPDH antibodies as indicated. The amount of GAPDH protein from each crude cell extract was used as loading control. Colony formation (e) and migration/invasion ability (f) of Bmi-1-overexpressing and control-GFP-expressing HNSCC-ALDH1^−^ were analyzed. *P < .05. Data shown here are the mean ± SD of three experiments.

To further investigate the role of Bmi-1 in maintaining the biological properties of HNSCC-ALDH1^+^, we used a loss-of-function approach, in which Bmi-1 was knocked down by small hairpin RNA (shRNA) in HNSCC-ALDH1^+^ cells. Stable knockdown of Bmi-1 in HNSCC-ALDH1^+^ cells was achieved by transduction with lentivirus that expressed shRNA targeting Bmi-1 (sh-Bmi-1). Lentivirus that expressed shRNA targeted against luciferase (sh-Luc.) was used as a control. Western blot analysis confirmed that shBmi-1 repressed Bmi-1 protein expression in HNSCC-ALDH1^+^ cells (Figure 2(a)). Importantly, silencing Bmi-1 expression led to downregulation of Snail and ALDH1 expression (Figure 2(a)). Additionally, our results showed that silencing of Bmi-1 in HNSCC-ALDH1^+^ cells inhibited the ability of the cells to form colonies on soft agar (Figure 2(b)) and migrate/invade (Figure 2(c)).

### 3.3. Overexpression of Bmi-1 in HNSCC-ALDH1^−^ Cells Enhances Tumorigenic Properties by Upregulating Snail

To evaluate whether overexpression of Bmi-1 could enhance the tumorigenic properties of HNSCC-ALDH1^−^ cells, we generated stable Bmi-1-overexpressing (Bmi-1Over) HNSCCs using lentiviral transduction (Figure 2(d)). Total proteins from HNSCC-ALDH1**^−^** overexpressing Bmi-1 exhibited elevated expression of Snail and ALDH1 (Figure 2(d)). In addition, overexpression of Bmi-1 significantly increased soft agar colony formation (Figure 2(e)), and migration/invasion of HNSCC-ALDH^−^ cells (Figure 2(f)). Taken together, our results suggest that Bmi-1 modulates the *in vitro* tumorigenic properties in HNSCC-ALDH1^+^ or ALDH1^−^ cells by regulating Snail.

### 3.4. Overexpression of Bmi-1 in HNSCC-ALDH1^−^ Cells Promotes Stemness Properties

**Figure 3:**
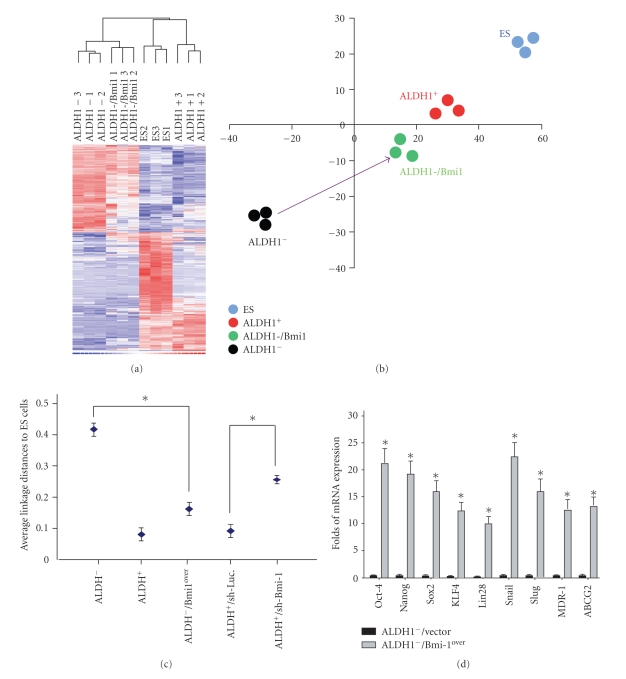
Stemness properties were enhanced in HNSCC-ALDH1^−^ cells when Bmi-1 was overexpressed. (a) Gene expression microarray analysis (Gene tree) for altered genes differentially expressed in Bmi-1-overexpressing HNSCC-ALDH1^−^ cells compared to HNSCC-ALDH1^−^ cells by a hierarchy heat map. The time dependent changes of altered genes are presented on a log scale of expression values provided by GeneSpring GX software. (b) Principle component analysis (PCA) demonstrated that overexpression of Bmi-1 in HNSCC-ALDH1^−^ cells could enhance the gene signature of embryonic stem cells (ESCs) in HNSCC-ALDH1^−^ cells. (c) Multidimensional scaling analysis. Average lineage transcriptome distances between HNSCC-ALDH1^+^, HNSCC-ALDH1^−^, HNSCC-ALDH1^+^/sh-Bmi-1, and HNSCC-ALDH1^−^/Bmi1^over^ cells. *P < .05. (d) Transcripts of Oct-4, Nanog, Sox2, KLF4, Lin28, Snail, Slug, MDR-1, and ABCG2 in HNSCC-ALDH1^−^ and HNSCC-ALDH1^−^/Bmi-1^over^ cells (*P < .05: ALDH1^−^ versus Bmi-1-overexpressing ALDH1^−^).

To explore molecules governing stemness and tumorigenicity in HNSCC-CD44^−^ALDH1^−^ cells treated with Bmi1-overexpressing lentivirus, we examined their transcriptome profile using gene expression microarray analysis (Figure 3(a)). Principle component analysis (PCA) further showed that the transcriptome profile of HNSCC-ALDH1^−^ cells overexpressing Bmi-1 demonstrated higher expression levels of embryonic stem cells (ESCs) transcriptomes (Table 3 and Figure 3(b)). Multidimensional scaling analysis further demonstrated that HNSCC-ALDH1^+^ cells and HNSCC-ALDH1^−^ cells overexpressing Bmi-1 are more similar to ESCs than HNSCC-ALDH1^−^ cells (*P < .05; Figure 3(c)). To validate the microarray analysis results, real-time PCR was performed to confirm that the mRNA expression levels of the embryonic genes (Oct-4, Nanog, Sox2, KLF4, and Lin28), EMT-related genes (Snail and Slug), and drug-resistant-related genes (MDR-1 and ABCG2) in Bmi-1-overexpressing ALDH1^−^ cells were significantly higher than those in ALDH1^−^ cells (*P < .05; Table 2 and Figure 3(d)). 

### 3.5. Elevation of In Vivo Tumor Growth, Metastatic Activity, and Radioresistance in HNSCC-ALDH1^−^ Cells by Overexpression of Bmi-1

**Figure 4:**
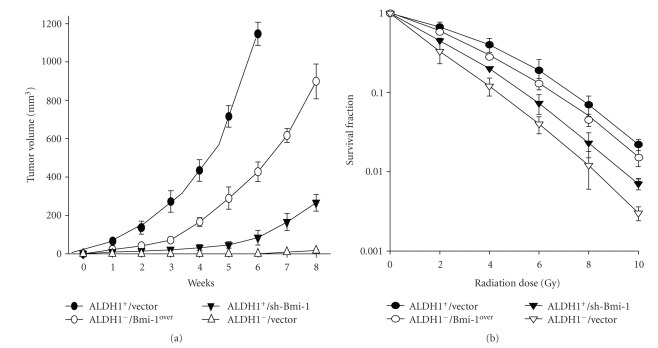
Determination of the role of Bmi-1 on *in vivo* tumor growth and radioresistance in HNSCC-ALDH1^+^cells. (a) Tumor volume was measured after injection of either HNSCC-ALDH1^+^, sh-Bmi-1 treated HNSCC-ALDH1^+^, HNSCC-ALDH1**^−^**, or Bmi-1-overexpressing HNSCC- ALDH1**^−^** cells into the neck of SCID mice. Error bars correspond to SD. (b) To determine the radiation effect on the cell survival rate, an ionizing radiation (IR) dose from 0 to 10Gy was used to treated with ALDH1^+^/vector, ALDH1^+^/sh-Bmi-1, ALDH1**^−^** /vector, or Bmi-1-overexpressing HNSCC- ALDH1**^−^** HNSCC cells.

**Figure 5:**
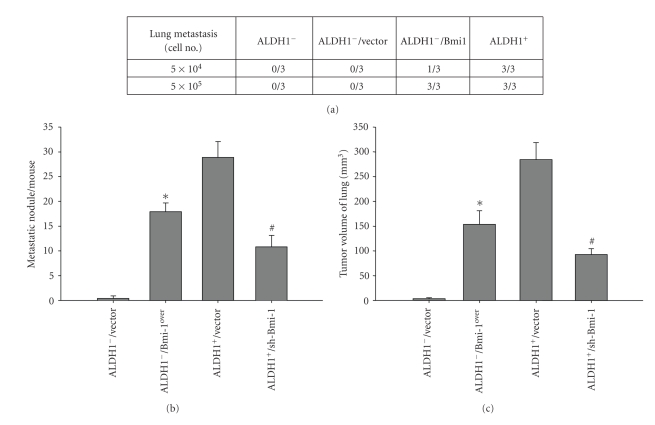
Elimination of metastatic activity in HNSCC-ALDH1^+^cells treated with shBmi-1. (a) Summary of the *in vivo* metastasis ability of different numbers of HNSCC-ALDH1^+^, sh-Bmi-1 treated HNSCC-ALDH1^+^, HNSCC- ALDH1**^−^**, or Bmi-1-overexpressing HNSCC- ALDH1**^−^** cells examined by xenotransplantation analysis. (b) The average numbers of metastatic foci (*left panel*) and total weight (*right panel*) in the lungs of mice treated with either HNSCC-ALDH1^+^, sh-Bmi-1 treated HNSCC-ALDH1^+^, HNSCC- ALDH1**^−^**, or Bmi-1-overexpressing HNSCC-ALDH1**^−^**cells are shown. (*P < .05: ALDH1^−^ versus Bmi-1-overexpressing ALDH1^−^; ^#^ *P* < .05: ALDH1^+^ versus shBmi-1 treated HNSCC-ALDH1^+^).

We next sought to determine if Bmi-1 expression could modulate the *in vivo* tumor initiating activity in immunocompromised nude mice. To monitor the *in vivo* growth of ALDH1^+^, ALDH1^−^, and Bmi-1-overexpressing ALDH1^−^ cells, these cells were transfected using a lentivector combined with the green fluorescent protein gene (GFP) and followed by *in vivo *GFP imaging system. Firstly, the results showed that 1 × 10^4^ALDH1^−^ cells did not induce tumor formation in nude mice, but 1000 ALDH1^+^ cells generated visible tumors 6 weeks after injection (Table 1). In contrast to ALDH1^−^ cells, one of three (33.3%) nude mice was detected with the tumor formation after 6-week transplantation of 3000 Bmi-1-overexpressing ALDH1^−^ cells. Furthermore, tumor volumes in HNSCC-ALDH1^+^ transplanted mice were significantly decreased when mice were treated with sh-Bmi-1 (Table 1; Figure 4(a)). Overexpression of Bmi-1 enhanced *in vivo* tumor growth in HNSCC-ALDH1^−^ (Table 1; Figure 4(a)). Furthermore, we investigated the role of Bmi-1 in the radio sensitivity of HNSCC-ALDH1^−^ and HNSCC-ALDH1^+^ treated with sh-Bmi-1 and Bmi-1 overexpressing. An ionizing radiation (IR) dose of 0 to 10 Gy was applied to these cells, and HNSCC-ALDH1^+^ cells showed greater radioresistance than the ALDH1^−^ cells (*P* < .05; Figure 4(b)). Knockdown of BMI-1 in ALDH1^+^ cells results in significant inhibition of radioresistance while overexpression of BMI-1 in ALDH- cells promotes radioresistant properties (*P* < .05; Figure 4(b)). Moreover, to confirm that Bmi-1 is crucial for metastasis *in vivo*, mice were injected with different numbers of ALDH1^+^, ALDH1^+^/sh-Bmi-1, ALDH1^−^/Bmi-1over or control GFP-expressing ALDH1^−^ cells. 5x105 Bmi-1-overexpressing ALDH1^−^ cells significantly increased local invasion, distant metastasis to the lungs and tumor size compared with control ALDH1^−^ cells (Figures 5(a) and 5(b)). In addition, silencing Bmi-1 in ALDH1^+^ cells effectively reduced the number of lung metastases and tumor size* in vivo *(Figures 5(a) and 5(b)). Taken together, our results reveal a crucial role for Bmi-1 signaling in the maintenance of *in vivo* tumorigenicity and metastasis of HNSCC-ALDH1^+^ and -ALDH1^−^ cells.

### 3.6. Coexpression of Bmi-1, Snail, and ALDH1 in HNSCC Tissues Correlates with Poor Overall Survival Rate of HNSCC Patients

**Figure 6:**
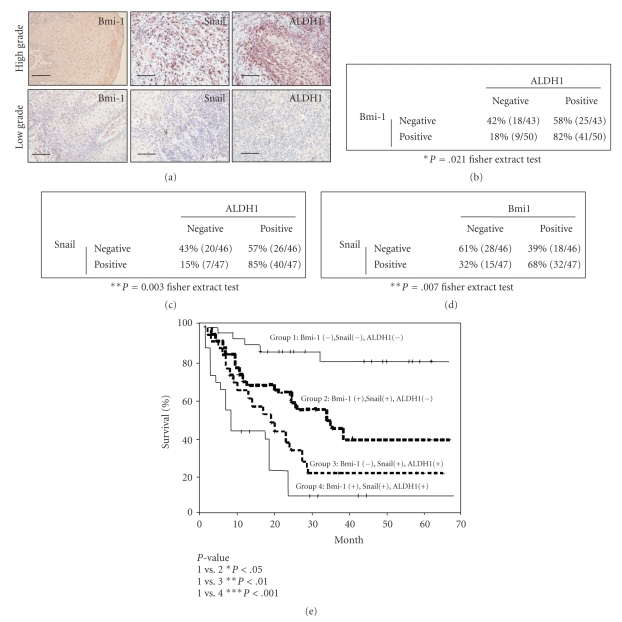
Coexpression of Bmi-1, Snail, and ALDH1 in HNSCC patient specimen and prediction of survival of the HNSCC patients. (a) Representative pictures of triple positive (*upper panel*) and triple negative (*lower panel*) HNSCC cases. Coexpression of Bmi-1 and ALDH1 (b), Bmi-1 and Snail (c) or Snail and Bmi-1 (d) of 93 HNSCC patient samples were examined immunohistochemically. (e) Kaplan-Meier analysis of overall survival of HNSCC patients according to expression of ALDH1 (+) Bmi-1 (+) Snail (+), ALDH1 (+) Bmi-1 (+) Snail (−), ALDH1 (−) Bmi-1 (+) Snail (+) or ALDH1 (−) Bmi-1 (−) Snail (−). (*, *P* < .05; **, *P* < .01; ***, *P* < .001).

Elevated Snail protein expression in HNSCC is correlated with the development of metastasis and poor survival [37]. Elevated expression of ALDH1 also correlates with poor prognosis for HNSCC patients [13]. To investigate whether there is a positive correlation between Bmi-1, Snail, and ALDH1 in head and neck cancers, we studied the expression of Bmi-1, Snail, and ALDH1 by immunohistochemical (IHC) staining of a panel of specimens array from 93 HNSCC patients. The IHC results showed that elevated expression of Bmi-1, Snail, and ALDH1 was positively associated with high-grade, poorly differentiated HNSCC (Figure 6(a)). Our results also showed a significant positive correlation between ALDH-1, Bmi-1 (Figure 6(b)); ALDH-1 and Snail (Figure 6(c)); Bmi-1 and Snail (Figure 6(d)) in HNSCC tissues. This is consistent with previous studies that reported that HNSCC-ALDH1^+^ cells have elevated Bmi-1 and Snail expression [13, 38]. To determine the prognostic significance of Bmi-1, Snail, and ALDH1 coexpression in patients with HNSCC, Kaplan-Meier survival analysis was performed. Patients who were triple positive for Bmi-1, Snail, and ALDH1 were predicted to have the worst survival rate compared with other head and neck cancer patients (Figure 6(e); Bmi-1^+^/Snail^+^/ALDH1^+^ versus other groups). Overall, these data indicate that expression of Bmi-1, Snail, and ALDH1 in HNSCC patients could be a critical factor in predicting disease progression and clinical outcomes.

## 4. Discussion

A recent study demonstrated that Bmi-1 mRNA and protein overexpressed in a subpopulation of tumor initiating cells in CD44+ HNSCC, which possessed self-renewal and tumor formation ability [39]. Zhang et al. also reported that there are side populations of oral squamous cell carcinomas that express high levels of ABCG2, ABCB1, CD44, Oct-4, Bmi-1, NSPc1, and CK19 [28]. Our previous work showed that HNSCC-ALDH1+ cells have high levels of Bmi-1. The ability to self-renew and radiochemoresistance were significantly suppressed in Bmi-1-silenced HNSCC-ALDH1+ cells [38]. Using microarray, western-blotting, and immunofluorescent assays, Chen et al. confirmed that ALDH1+-lineage cells underwent epithelial-mesenchymal transition (EMT) and endogenously co-expressed Snail [13]. In the current study, our data demonstrated that HNSCC-ALDH1^+^ cells had high levels of Bmi-1, at both the mRNA and protein levels (Figure 2). Using a lentiviral vector expressing shRNA targeting Bmi-1, we observed that the level of ALDH1 expression and tumorigenic properties of HNSCC-ALDH1^+^ could be down-regulated by knockdown of Bmi-1 (Figure 2). Importantly, overexpression of Bmi-1 could turn HNSCC-ALDH1^−^ into cancer stem cell-like HNSCC-ALDH1^+^ cells (Figure 3). Consistent with these findings, the immunohistochemical survey of 93 HNSCC patient tissues showed a positive correlation between expression of Bmi-1, Snail, or ALDH1 and tumor stage (Figure 6). Similar results were noted in other malignancies [40]. Kaplan-Meier analysis demonstrated that patients expressing Bmi-1, Snail, and ALDH1 were predicted to have the worst survival prognosis of HNSCC patients (Figure 6(e)). However, a recent study showed a significant correlation between negative Bmi-1 protein expression and the recurrence of tongue cancer. Their results showed Snail and c-myc expression did not correlate with prognosis [41]. The divergence from our results may be due to the different pathophysiology of HNSCC. Most HNSCC patients in Taiwan consume alcohol, chew betel quid and smoke cigarettes. Tongue cancer patients, especially female tongue cancer patients, usually do not have these habits [3]. The close relationship between tongue cancer and human papillomavirus has been explored by many researchers [42–45]. The correlation between cancer stem cells and the virus infection remains to be discovered.

The prognosis of HNSCC patients with distant metastases in the lung, liver, and bone is very poor [3, 46]. In this study, we found that Bmi-1 can regulate Snail and ALDH1; change the EMT-related genotypes of the ALDH1^−^ cells; and modulate distant lung metastases (Figure 5). Distant metastases have been reported to be associated with Bmi-1 expression in breast cancer [47–49], melanoma [50], gastric cancer [51], and colon cancer [30]. Microarray analysis revealed that eleven gene signatures, which were correlated to the Bmi-1-driven pathway, were closely related to distant lung metastases [40]. Bmi-1 is the target gene of SALL4 in human hematopoietic as well as leukemic cells and is down-regulated if SALL4 is knocked down by the siRNA in the HL-60 leukemia cell line [52, 53]. Recently, researchers employed microRNA profiling to gain insight into the role of Bmi-1 in regulating EMT. Overexpression of miR-200c decreased Bmi-1 expression in breast cancer stem cells (BCSCs) and inhibited the formation of mammary ducts as well as tumors by normal mammary stem cells and BCSCs [54]. Bhattacharya et al. found that miR-15a and miR-16 directly targeted the Bmi-1 3′ untranslated region and correlated with Bmi-1 protein levels in ovarian cancer patients and cell lines [55]. Further research effort is needed in this area. Together, our research shows that the Bmi-1 signaling pathways play a major role in the maintenance of stemness and the metastatic ability of HNSCC-CSC by regulating of Snail expression. Additionally, we demonstrate coexpression of Bmi-1, Snail, and ALDH1 in HNSCC patients was positively correlated with tumor grade and the worst prognosis.

## Supplementary Material

Figure 1. (a) Cell morphology in ALDH1+ HNSCC cells under specific serum free medium and 10% serum (right panel). Epithelial differentiation marker, CK18 positive cells in ALDH1+ HNSCC cells under specific serum free medium and 10% serum (right panel). (b) In vivo self-renewal ability of HNSCC-ALDH1+ cells.

## Figures and Tables

**Table 1:** Case description, tumorigenic characteristics and treatment effects of ALDH1^+^ and ALDH1^−^ HNSCC.

				Number of cells injected				
Case	Age/Sex	ALDH^+^ (%)	Spheres	Parental	ALDH1^+^	ALDH1^+^ (Sh-Bmi 1)	ALDH1^−^	ALDH1^−^ (Bmi 1Over)
			Formation					
1	71/ M	44.2	Yes	1,000 (0/3)	1,000 (1/3)	1,000 (0/3)	1,000 (0/3)	1,000 (1/3)
				3,000 (0/3)	3,000 (3/3)	3,000 (0/3)	3,000 (0/3)	3,000 (1/3)
				10,000 (1/3)	10,000 (3/3)	10,000 (2/3)	10,000 (1/3)	10,000 (2/3)

2	73/ F	24.7	Yes	1,000 (0/3)	1,000 (1/3)	1,000 (1/3)	1,000 (0/3)	1,000 (0/3)
				3,000 (0/3)	3,000 (2/3)	3,000 (2/3)	3,000 (0/3)	3,000 (1/3)
				10,000 (2/3)	10,000 (2/3)	10,000 (2/3)	10,000 (0/3)	10,000 (2/3)

3	61 / F	8.6	Yes	1,000 (0/3)	1,000 (1/3)	1,000 (1/3)	1,000 (0/3)	1,000 (0/3)
				3,000 (0/3)	3,000 (3/3)	3,000 (1/3)	3,000 (0/3)	3,000 (2/3)
				10,000 (0/3)	10,000 (3/3)	10,000 (2/3)	10,000 (0/3)	10,000 (3/3)

4	71 / M	1.2	Yes	1,000 (0/3)	1,000 (0/3)	1,000 (0/3)	1,000 (0/3)	1,000 (0/3)
				3,000 (0/3)	3,000 (3/3)	3,000 (0/3)	3,000 (0/3)	3,000 (2/3)
				10,000 (0/3)	10,000 (3/3)	10,000 (2/3)	10,000 (0/3)	10,000 (3/3)

5	69 / M	19.2	Yes	1,000 (0/3)	1,000 (1/3)	1,000 (0/3)	1,000 (0/3)	1,000 (0/3)
				3,000 (0/3)	3,000 (3/3)	3,000 (1/3)	3,000 (0/3)	3,000 (2/3)
				10,000 (1/3)	10,000 (3/3)	10,000 (2/3)	10,000 (0/3)	10,000 (3/3)

6	72 / M	5.5	Yes	1,000 (0/3)	1,000 (1/3)	1,000 (0/3)	1,000 (0/3)	1,000 (0/3)
				3,000 (0/3)	3,000 (3/3)	3,000 (1/3)	3,000 (0/3)	3,000 (2/3)
				10,000 (0/3)	10,000 (3/3)	10,000 (1/3)	10,000 (0/3)	10,000 (2/3)

ALDH1^+^: ALDH1-positive HNSCC cells; ALDH1^−^: ALDH1-negative HNSCC cells.

ALDH1^+^ or ALDH1^+^cells were injected into neck of SCID mice.

**Table 2:** The sequences for the primers of quantitative RT-PCR.

Gene(Accession No.)	Primer Sequence (5′ to 3′)	Product size (bp)	Tm (°C)
Oct-4(NM_002701)	F: GTGGAGAGCAACTCCGATG	86	60
	R: TGCTCCAGCTTCTCCTTCTC		

Nanog(NM_024865)	F: ATTCAGGACAGCCCTGATTCTTC	76	60
	R: TTTTTGCGACACTCTTCTCTGC		

SOX-2(NM_003106)	F: CGAGTGGAAACTTTTGTCGGA	74	60
	R: TGTGCAGCGCTCGCAG		

Musashi(NM_002442)	F: TCCCTCGGCGAGCACA	64	60
	R: GACAGCCCCCCCACAAA		

c-Myc(NM_002467)	F: GGAACGAGCTAAAACGGAGCT	71	55
	R: GGCCTTTTCATTGTTTTCCAACT		

*β*-catenin(NM_001904)	F: CCAGCCGACACCAAGAAG	130	55
	R: CGAATCAATCCAACAGTAGCC		

Bmi1(NM_ 005180)	F*：*AAATGCTGGAGAACTGGAAAG	124	50
	R*：*CTGTGGATGAGGAGACTGC		

Nestin(NM_006617)	F: AGGAGGAGTTGGGTTCTG	112	50
	R: GGAGTGGAGTCTGGAAGG		

Snail(NM_005985)	F:GCTGCCAATGCTCATCTGGGACTCT	300	55
	R: TTGAAGGGCTTTCGAGCCTGGAGAT		

Slug(NM_003068)	F: GTGATTATTTCCCCGTATCTCTAT	292	50
	R: CAATGGCATGGGGGTCTGAAAG		

MDR-1 (NM_000927)	F: TGGCAAAGAAATAAAGCGACTGA	76	60
	R: CAGGATGGGCTCCTGGG		

MRP-1(X60111)	F: GCTTCCTCTTGGTGATATTCG	176	50
	R: GCAGTTCAACGCATAGTGG		

ABCG2(NM_004827)	F: CATGTACTGGCGAAGAATATTTGGT	74	60
	R: CACGTGATTCTTCCACAAGCC		

GAPDH(NM_002046)	F: CATCATCCCTGCCTCTACTG	180	60
	R: GCCTGCTTCACCACCTTC		

**Table 3:** The expression profiling of up-regulated genes in ALDH1^−^/Bmi1-overexpressed as compared to ALDH1^−^ HNSCC.

Probe set ID	Gene symbol	Gene title
217757_at	A2M	alpha-2-macroglobulin
209459_s_at	ABAT	4-aminobutyrate aminotransferase
213353_at	ABCA5	ATP-binding cassette, sub-family A (ABC1), member 5
209993_at	ABCB1(MDR1)	ATP-binding cassette, sub-family B (MDR/TAP), member 1
214033_at	ABCC6	ATP-binding cassette, sub-family C (CFTR/MRP), member 6
210246_s_at	ABCC8	ATP-binding cassette, sub-family C (CFTR/MRP), member 8
204567_s_at	ABCG1	ATP-binding cassette, sub-family G (WHITE), member 1
209735_at	ABCG2	ATP-binding cassette, sub-family G (WHITE), member 2
204151_x_at	AKR1C1	aldo-keto reductase family 1, member C1 (dihydrodiol dehydrogenase 1; 20-alpha (3-alpha)-hydroxysteroid dehydrogenase)
209699_x_at	AKR1C2	aldo-keto reductase family 1, member C2 (dihydrodiol dehydrogenase 2; bile acid binding protein; 3-alpha hydroxysteroid dehydrogenase, type III)
212224_at	ALDH1A1	aldehyde dehydrogenase 1 family, member A1
204446_s_at	ALOX5	arachidonate 5-lipoxygenase
205216_s_at	APOH	apolipoprotein H (beta-2-glycoprotein I)
39248_at	AQP3	aquaporin 3 (Gill blood group)
218501_at	ARHGEF3	Rho guanine nucleotide exchange factor (GEF) 3
219087_at	ASPN	Aspirin
201242_s_at	ATP1B1	ATPase, Na+/K+ transporting, beta 1 polypeptide
200921_s_at	BTG1	B-cell translocation gene 1, anti-proliferative
228067_at	C2orf55	chromosome 2 open reading frame 55
206488_s_at	CD36	CD36 molecule (thrombospondin receptor)
208783_s_at	CD46	CD46 molecule, complement regulatory protein
1553970_s_at	CEL	carboxyl ester lipase (bile salt-stimulated lipase)
203854_at	CFI	complement factor I
205043_at	CFTR	cystic fibrosis transmembrane conductance regulator (ATP-binding cassette sub-family C, member 7)
204260_at	CHGB	chromogranin B (secretogranin 1)
221188_s_at	CIDEB	cell death-inducing DFFA-like effector b
203953_s_at	CLDN3	claudin 3
221042_s_at	CLMN	calmin (calponin-like, transmembrane)
1567081_x_at	CLN6	ceroid-lipofuscinosis, neuronal 6, late infantile, variant
208791_at	CLU	Clusterin
229831_at	CNTN3	contactin 3 (plasmacytoma associated)
205615_at	CPA1	carboxypeptidase A1 (pancreatic)
206212_at	CPA2	carboxypeptidase A2 (pancreatic)
205509_at	CPB1	carboxypeptidase B1 (tissue)
201117_s_at	CPE	carboxypeptidase E
224829_at	CPEB4	cytoplasmic polyadenylation element binding protein 4
204920_at	CPS1	carbamoyl-phosphate synthetase 1, mitochondrial
201990_s_at	CREBL2	cAMP responsive element binding protein-like 2
205971_s_at	CTRB1 /// CTRB2	chymotrypsinogen B1 /// chymotrypsinogen B2
214411_x_at	CTRB2	chymotrypsinogen B2
209774_x_at	CXCL2	chemokine (C-X-C motif) ligand 2
205765_at	CYP3A5	cytochrome P450, family 3, subfamily A, polypeptide 5
228391_at	CYP4V2	cytochrome P450, family 4, subfamily V, polypeptide 2
228739_at	CYS1	cystin 1
222925_at	DCDC2	doublecortin domain containing 2
205311_at	DDC	dopa decarboxylase (aromatic L-amino acid decarboxylase)
210397_at	DEFB1	defensin, beta 1
221081_s_at	DENND2D	DENN/MADD domain containing 2D
214787_at	DENND4A	DENN/MADD domain containing 4A
205684_s_at	DENND4C	DENN/MADD domain containing 4C
214079_at	DHRS2	dehydrogenase/reductase (SDR family) member 2
222850_s_at	DNAJB14	DnaJ (Hsp40) homolog, subfamily B, member 14
225415_at	DTX3L	deltex 3-like (Drosophila)
225645_at	EHF	Ets homologous factor
210080_x_at	ELA3A	elastase 3A, pancreatic
201510_at	ELF3	E74-like factor 3 (ets domain transcription factor, epithelial-specific)
206191_at	ENTPD3	ectonucleoside triphosphate diphosphohydrolase 3
220012_at	ERO1LB	ERO1-like beta (S. cerevisiae)
210103_s_at	FOXA2	forkhead box A2
235201_at	FOXP2	forkhead box P2
226847_at	FST	Follistatin
205674_x_at	FXYD2	FXYD domain containing ion transport regulator 2
205890_s_at	GABBR1 /// UBD	gamma-aminobutyric acid (GABA) B receptor, 1 /// ubiquitin D
205848_at	GAS2	growth arrest-specific 2
216733_s_at	GATM	glycine amidinotransferase (L-arginine:glycine amidinotransferase)
204965_at	GC	group-specific component (vitamin D binding protein)
219508_at	GCNT3	glucosaminyl (N-acetyl) transferase 3, mucin type
225853_at	GNPNAT1	glucosamine-phosphate N-acetyltransferase 1
212950_at	GPR116	G protein-coupled receptor 116
212070_at	GPR56	G protein-coupled receptor 56
203924_at	GSTA1	glutathione S-transferase A1
221942_s_at	GUCY1A3	guanylate cyclase 1, soluble, alpha 3
228697_at	HINT3	histidine triad nucleotide binding protein 3
209558_s_at	HIP1R	huntingtin interacting protein 1 related
207062_at	IAPP	islet amyloid polypeptide
213620_s_at	ICAM2	intercellular adhesion molecule 2
203828_s_at	IL32	interleukin 32
205945_at	IL6R	interleukin 6 receptor
206598_at	INS	Insulin
226535_at	ITGB6	integrin, beta 6
226189_at	ITGB8	integrin, beta 8
210078_s_at	KCNAB1	potassium voltage-gated channel, shaker-related subfamily, beta member 1
219564_at	KCNJ16	potassium inwardly-rectifying channel, subfamily J, member 16
205303_at	KCNJ8	potassium inwardly-rectifying channel, subfamily J, member 8
212531_at	LCN2	lipocalin 2
235970_at	LCORL	ligand dependent nuclear receptor corepressor-like
1554006_a_at	LLGL2	lethal giant larvae homolog 2 (Drosophila)
225996_at	LONRF2	LON peptidase N-terminal domain and ring finger 2
242931_at	LONRF3	LON peptidase N-terminal domain and ring finger 3
226748_at	LYSMD2	LysM, putative peptidoglycan-binding, domain containing 2
213975_s_at	LYZ	lysozyme (renal amyloidosis)
222670_s_at	MAFB	v-maf musculoaponeurotic fibrosarcoma oncogene homolog B (avian)
223577_x_at	MALAT1	metastasis associated lung adenocarcinoma transcript 1 (non-protein coding)
220945_x_at	MANSC1	MANSC domain containing 1
204388_s_at	MAOA	monoamine oxidase A
235077_at	MEG3	maternally expressed 3
229254_at	MFSD4	major facilitator superfamily domain containing 4
219797_at	MGAT4A	mannosyl (alpha-1,3-)-glycoprotein beta-1,4-N-acetylglucosaminyltransferase, isozyme A
204259_at	MMP7	matrix metallopeptidase 7 (matrilysin, uterine)
227747_at	MPZL3	myelin protein zero-like 3
204438_at	MRC1 /// MRC1L1	mannose receptor, C type 1 /// mannose receptor, C type 1-like 1
203037_s_at	MTSS1	metastasis suppressor 1
212093_s_at	MTUS1	mitochondrial tumor suppressor 1
213693_s_at	MUC1	mucin 1, cell surface associated
213375_s_at	N4BP2L1	NEDD4 binding protein 2-like 1
220184_at	NANOG	Nanog homeobox
209107_x_at	NCOA1	nuclear receptor coactivator 1
1556057_s_at	NEUROD1	neurogenic differentiation 1
206915_at	NKX2-2	NK2 homeobox 2
225911_at	NPNT	Nephronectin
205259_at	NR3C2	nuclear receptor subfamily 3, group C, member 2
212768_s_at	OLFM4	olfactomedin 4
203845_at	PCAF	p300/CBP-associated factor
240317_at	PCDHB4	protocadherin beta 4
212593_s_at	PDCD4	programmed cell death 4 (neoplastic transformation inhibitor)
213228_at	PDE8B	phosphodiesterase 8B
225207_at	PDK4	pyruvate dehydrogenase kinase, isozyme 4
205380_at	PDZK1	PDZ domain containing 1
1553589_a_at	PDZK1IP1	PDZK1 interacting protein 1
226459_at	PIK3AP1	phosphoinositide-3-kinase adaptor protein 1
220954_s_at	PILRB	paired immunoglobulin-like type 2 receptor beta
219584_at	PLA1A	phospholipase A1 member A
206311_s_at	PLA2G1B	phospholipase A2, group IB (pancreas)
221529_s_at	PLVAP	plasmalemma vesicle associated protein
205912_at	PNLIP	pancreatic lipase
211766_s_at	PNLIPRP2	pancreatic lipase-related protein 2
208286_x_at	POU5F1(Oct4)	POU class 5 homeobox 1 /// POU class 5 homeobox 1B /// POU class 5 homeobox 1 pseudogene 3 /// POU class 5 homeobox 1 pseudogene 4
228469_at	PPID	Peptidylprolyl isomerase D (cyclophilin D)
210670_at	PPY	pancreatic polypeptide
242482_at	PRKAR1A	protein kinase, cAMP-dependent, regulatory, type I, alpha (tissue specific extinguisher 1)
227629_at	PRLR	Prolactin receptor
228656_at	PROX1	prospero homeobox 1
205869_at	PRSS1	protease, serine, 1 (trypsin 1)
205402_x_at	PRSS2	protease, serine, 2 (trypsin 2)
213421_x_at	PRSS3	protease, serine, 3
203317_at	PSD4	pleckstrin and Sec7 domain containing 4
203029_s_at	PTPRN2	protein tyrosine phosphatase, receptor type, N polypeptide 2
219562_at	RAB26	RAB26, member RAS oncogene family
226436_at	RASSF4	Ras association (RalGDS/AF-6) domain family member 4
223322_at	RASSF5	Ras association (RalGDS/AF-6) domain family member 5
235638_at	RASSF6	Ras association (RalGDS/AF-6) domain family member 6
204364_s_at	REEP1	receptor accessory protein 1
209752_at	REG1A	regenerating islet-derived 1 alpha (pancreatic stone protein, pancreatic thread protein)
205886_at	REG1B	regenerating islet-derived 1 beta (pancreatic stone protein, pancreatic thread protein)
205815_at	REG3A	regenerating islet-derived 3 alpha
1554003_at	RGNEF	Rho-guanine nucleotide exchange factor
219263_at	RNF128	ring finger protein 128
221614_s_at	RPH3AL	rabphilin 3A-like (without C2 domains)
213939_s_at	RUFY3	RUN and FYVE domain containing 3
210592_s_at	SAT1	spermidine/spermine N1-acetyltransferase 1
203408_s_at	SATB1	SATB homeobox 1
204035_at	SCG2	secretogranin II (chromogranin C)
205697_at	SCGN	secretagogin, EF-hand calcium binding protein
229620_at	SEPP1	Selenoprotein P, plasma, 1
202833_s_at	SERPINA1	serpin peptidase inhibitor, clade A (alpha-1 antiproteinase, antitrypsin), member 1
202376_at	SERPINA3	serpin peptidase inhibitor, clade A (alpha-1 antiproteinase, antitrypsin), member 3
209443_at	SERPINA5	serpin peptidase inhibitor, clade A (alpha-1 antiproteinase, antitrypsin), member 5
213572_s_at	SERPINB1	serpin peptidase inhibitor, clade B (ovalbumin), member 1
227627_at	SGK3	serum/glucocorticoid regulated kinase family, member 3
219256_s_at	SH3TC1	SH3 domain and tetratricopeptide repeats 1
204019_s_at	SH3YL1	SH3 domain containing, Ysc84-like 1 (S. cerevisiae)
213464_at	SHC2	SHC (Src homology 2 domain containing) transforming protein 2
205799_s_at	SLC3A1	solute carrier family 3 (cystine, dibasic and neutral amino acid transporters, activator of cystine, dibasic and neutral amino acid transport), member 1
223044_at	SLC40A1	solute carrier family 40 (iron-regulated transporter), member 1
228221_at	SLC44A3	solute carrier family 44, member 3
213139_at	SNAI2(Slug)	snail homolog 2 (Drosophila)
1560228_at	SNAI3(Snail)	snail homolog 3 (Drosophila)
213721_at	SOX2	SRY (sex determining region Y)-box 2
200795_at	SPARCL1	SPARC-like 1 (mast9, hevin)
206239_s_at	SPINK1	serine peptidase inhibitor, Kazal type 1
213921_at	SST	somatostatin
216905_s_at	ST14	suppression of tumorigenicity 14 (colon carcinoma)
230285_at	SVIP	small VCP/p97-interacting protein
227134_at	SYTL1	synaptotagmin-like 1
202286_s_at	TACSTD2	tumor-associated calcium signal transducer 2
205513_at	TCN1	transcobalamin I (vitamin B12 binding protein, R binder family)
203887_s_at	THBD	thrombomodulin
209937_at	TM4SF4	transmembrane 4 L six family member 4
226403_at	TMC4	transmembrane channel-like 4
223503_at	TMEM163	transmembrane protein 163
218345_at	TMEM176A	transmembrane protein 176A
220532_s_at	TMEM176B	transmembrane protein 176B
200847_s_at	TMEM66	transmembrane protein 66
202687_s_at	TNFSF10	tumor necrosis factor (ligand) superfamily, member 10
203824_at	TSPAN8	tetraspanin 8
229169_at	TTC18	tetratricopeptide repeat domain 18
209660_at	TTR	transthyretin (prealbumin, amyloidosis type I)
231008_at	UNC5CL	Unc-5 homolog C (C. elegans)-like
226344_at	ZMAT1	zinc finger, matrin type 1
206059_at	ZNF91	zinc finger protein 91
