# Supplementary figures and images for: Bmi-1 Regulates Snail Expression and Promotes Metastasis Ability in Head and Neck Squamous Cancer-Derived ALDH1 Positive Cells
Source: J Oncol. 2010 Sep 27;2011:609259. doi: 10.1155/2011/609259 (PMC2948925; doi:10.1155/2011/609259)

**(A)**

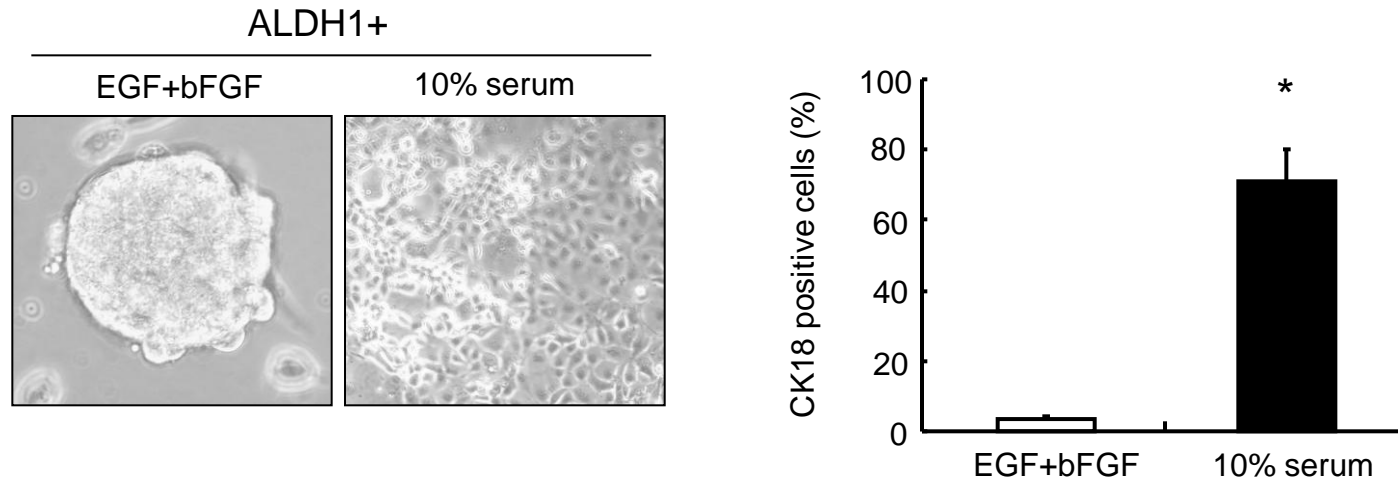

**(B)**

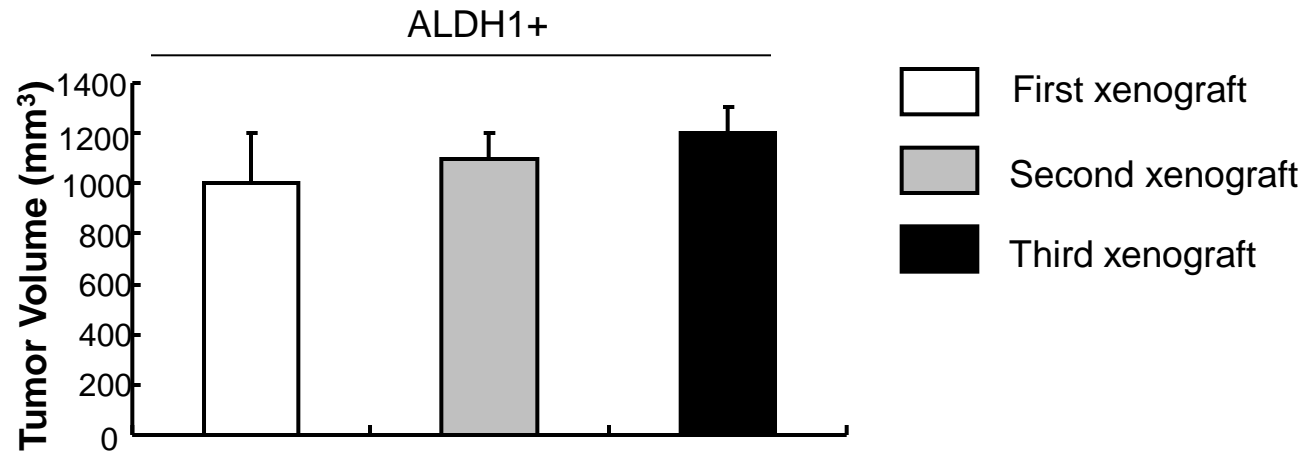

Supplement: Supplementary file 1 — Figure 1. (a) Cell morphology in ALDH1+ HNSCC cells under specific serum free medium and 10% serum (right panel). Epithelial differentiation marker, CK18 positive cells in ALDH1+ HNSCC cells under specific serum free medium and 10% serum (right panel). (b) In vivo self-renewal ability of HNSCC-ALDH1+ cells. [file 609259.f1.pdf]
